# Things you wanted to know about fungal extracellular vesicles (but were afraid to ask)

**DOI:** 10.1371/journal.pntd.0013038

**Published:** 2025-05-22

**Authors:** Flavia C. G. Reis, Marcio L. Rodrigues

**Affiliations:** 1 Instituto Carlos Chagas, Fundação Oswaldo Cruz (Fiocruz), Curitiba, Brazil; 2 Instituto de Microbiologia Paulo de Góes, Universidade Federal do Rio de Janeiro (UFRJ), Rio de Janeiro, Brazil; Albert Einstein College of Medicine, UNITED STATES OF AMERICA

## Introduction

Fungal diseases claim more than 2 million lives annually [1], yet this enormous impact on public health has not been historically accompanied by innovative solutions to combat lethal mycoses [2]. Invasive fungal infections primarily affect individuals facing socioeconomic restrictions, placing most of these conditions on the list of neglected diseases [3].

Neglected diseases persist due to multiple failures in science, the market, and public health [4]. Scientific failures occur when there is insufficient knowledge about the pathophysiology of infectious agents and the host response [4]. In this context, research on fungal extracellular vesicles (EVs) may help address these scientific gaps, as these structures influence the immune response against fungi, their pathogenic potential, cell-to-cell communication, drug resistance, and biofilm formation [5].

Fungal EVs were discovered in 2007 [6], and since then, research in this field has expanded exponentially [7]. However, many fundamental questions about fungal EVs remain unanswered, and several recent, comprehensive reviews have already explored these open questions in depth [8–12].

In this perspective, we do not aim to revisit previously discussed gaps in fungal EV research [13]. Instead, we seek to explore broader questions that may be of particular interest to newcomers and curious readers. For insights into the immunological functions of EVs, their role in pathogenesis, the mechanisms underlying their biogenesis, their impact on population-level communication, and drug resistance, we recommend the references listed in Table 1. We invite those intrigued by fungal EVs to reflect on the questions presented in the following sections.

**Table 1:** Illustration of the functions of fungal EVs.

Area of EV investigation	Organism(s)^*^ and references
Immunomodulation	*Cryptococcus neoformans* [37,46,50] *Aspergillus fumigatus* [56–58] *Aspergillus flavus* [23] *Candida albicans* [41–45,59,60] *Candida haemulonii* [49] *Malassezia furfur* [61] *Paracoccidioides brasiliensis* [17,47,48,51,53,62] *Candida auris* [63] *Talaromyces marneffei* [64]
Biogenesis	*C. neoformans* [38] *C. albicans* [65,66]
Drug resistance	*C. neoformans* [38] *C. auris* [67] *C. albicans* [66]
Cell-to-cell communication	*C. neoformans* and *Cryptococcus deuterogattii* [68] *C. albicans* [69–71] *A. fumigatus* and *P. brasiliensis* [71]
Biofilm formation	*C. auris*, *C. albicans*, *Candida tropicalis*, *Candida parapsilosis*, and *Candida glabrata* [66,69,72,73]

*Plant pathogens and the model yeast *Saccharomyces cerevisiae* were not included in this compilation. Notably, this is a functional illustration of fungal EVs, and additional related reports may be available in the literature.

## Are fungal EVs real or just artifacts?

They are real. This has been an important question in the field since fungal EVs were first described. Because EVs are nanosized (50–1000 nm) membranous particles, experimental approaches for their identification typically rely on nanoparticle detection combined with microscopic observation [14]. However, biological samples often contain various types of nanoparticles with bilayered membranes, which can be misidentified as EVs during nanoparticle tracking analysis and transmission electron microscopy, for instance. Examples of such particles include cell debris and organelles leaked from dead cells. Thus, it is a valid concern to question whether fungal EVs are contaminated with, or even correspond to, artifacts. This discussion has led to the development of several approaches to confirm that fungal EVs are the product of a physiological mechanism of molecular export. Dead fungal cells do not release particles with properties matching those of EVs [6]. Additionally, vesicles have been observed in association with the cell wall [6,15–17], as well as budding from the plasma membrane across the cell wall [16,18,19].

Compositional analyses of fungal EVs further demonstrated that they did not simply replicate the protein and RNA content of cellular extracts [20,21], supporting the idea that EV export is a regulated process. In summary, while questioning whether EV formation is a physiological event was an important step for the field, extensive experimental evidence from fungi and diverse organisms across different kingdoms of life strongly supports the notion that EV formation is an integral part of cellular physiology.

## Do all fungi produce EVs?

We still do not know, and this is a very difficult question to answer, as it is virtually impossible to test all fungi for EV production. For several years, EVs were exclusively characterized in the yeast forms of fungi [22], likely due to the ease of handling unicellular cultures. However, as EV isolation protocols became more refined, EV detection became possible in cultures of filamentous and dimorphic fungi, as well as in fungal spores [16,23–26].

As of early 2024, EV production has been reported in 32 fungal species [5], and to the best of our knowledge, no studies have yielded negative results when testing fungi for EV production. Therefore, while we cannot definitively state that all fungi produce EVs, the existing literature—along with the fact that EV production appears to be a universal phenomenon across all domains of life—suggests that this is a widespread physiological mechanism used by fungi for biomolecule export.

## Can we call fungal EVs exosomes?

No, we cannot. Historically, many scientists have referred to most small EVs as exosomes, making it a common term in the field of EV research. However, exosomes—EVs originating from the endosomal system—are just one of several subtypes within a diverse range of membrane vesicles [27]. They have unique characteristics, including specific components and biogenesis. Other types of EVs include ectosomes (which vary widely in size and originate from the plasma membrane), microvesicles (large EVs derived from the plasma membrane) [28], migrasomes (formed in migrating cells to mediate intercellular communication) [29], exophers (large, membrane-enclosed vesicles extruded from cells that can contain organelles) [30], and, as recently described, blebbisomes (organelle-rich EVs with cell-like properties) [31]. Finally, apoptosis can lead to the release of apoptotic exosome-like vesicles, a subtype of EVs secreted by apoptotic cells after caspase-3 activation [32]. The field of EV research has been constantly evolving and nomenclature changes frequently. For instance, two non-vesicular extracellular nanoparticles, exomeres and supermeres, have been discovered recently and are enriched in many cargoes previously ascribed to EVs [33].

**Fig 1:**
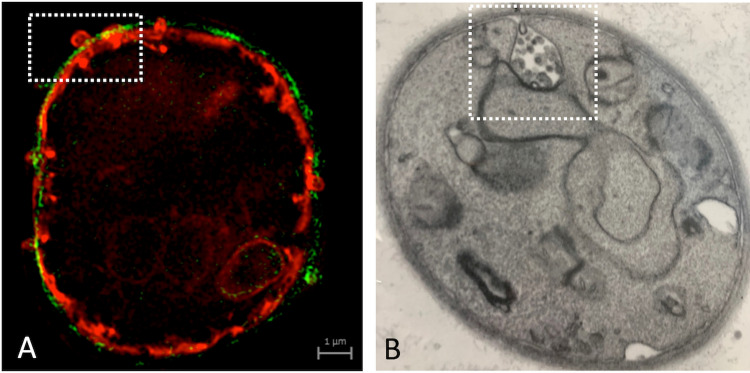
Visual illustration of possible mechanisms of EV biogenesis in fungi, including putative microvesicle formation in *Aspergillus fumigatus* conidia (A) and exosomes in *Cryptococcus neoformans* **(B)**. In panel A, the plasma membrane of an *A. fumigatus* conidium was stained in red with the dye DiI, while the cell wall was stained with an antibody raised to alpha glucan (green). The cell was examined by ultra-resolution fluorescence microscopy, revealing an event of plasma membrane budding across the cell wall in the boxed area. For experimental details, please refer to reference [16], from which this figure has been reproduced. Panel B depicts a TEM image of *C. neoformans*, with the boxed area highlighting a multivesicular body (MVB)-like structure, possibly in fusion with the plasma membrane, resulting in the release of intraluminal vesicles. This image is courtesy of Ana Caroline Colombo and Maurizio Del Poeta.

A large body of literature provides detailed definitions of all EV types. Notably, the International Society for Extracellular Vesicles (ISEV) generally recommends using “EVs” as the most appropriate term unless the subcellular origin is clearly demonstrated [28]. The subcellular origin of fungal EVs has not yet been established, although both molecular and microscopic evidence suggest they may originate from the endosomal compartment and/or the plasma membrane. In *Aspergillus fumigatus*, membrane budding and vesicle projections across the cell wall have been documented [16], aligning more closely with ectosomes or microvesicles (Fig 1A). In *Cryptococcus neoformans*, endosome-like compartments resembling multivesicular bodies have been observed fusing with the plasma membrane, suggesting that fungal EVs could correspond to exosomes [34,35], as illustrated in Fig 1B. However, since the molecular regulation of EV formation remains uncharacterized, we consider the ISEV’s recommendation appropriate, using “EVs” as the best and most accurate term for the fungal extracellular particles.

## Is working with fungal EVs easy?

Not always. For years, fungal EV analysis relied on protocols originally developed for isolating mammalian exosomes, adapting them to study vesicles exported by yeast forms of fungi. During the first decade of fungal EV research, fundamental aspects of EV production remained unresolved, including the optimal conditions for vesicle isolation, the cultivation time required to reach peak EV production, the identification of highly efficient EV-producing strains, comparisons of EV production across different morphological stages, and the development of protocols to separate EV subpopulations, among other parameters. As the concept of fungal EV production became more established, significant progress followed. For instance, in recent years, some laboratories have begun isolating EVs from fungal solid cultures [36,37]. While this approach may seem counterintuitive at first, the fluidity of the growth medium should not be a limiting factor, considering that EV production is an intrinsic part of fungal physiology. Indeed, fungal EVs can be successfully isolated from several species grown on solid media [36], offering the advantage of avoiding the large-volume centrifugation required in earlier protocols.

The field has also advanced through the optimization of high-throughput genetic screenings using mutant collections [38], the application of centrifugation-based gradients [39], and the use of nanoflow cytometry to identify EV surface markers [37]. Additionally, EVs have been detected during infections of plant cells [40].

The complexity of obtaining EVs is directly related to the ease of culturing fungal cells. To answer whether working with fungal EVs is easy—it largely depends on the fungal model being studied. However, there is no doubt that the process is significantly less challenging today than it was a decade ago.

## Do fungal EVs benefit the host?

Yes, under certain conditions. The past 2 years have seen undeniable progress in understanding how fungal EVs can enhance the host immune response. It has been recently demonstrated that *Candida albicans* EVs specifically activate type I interferon signaling in host cells via the cGAS-STING innate immune pathway [41]. In fact, fungal EVs effectively target innate immune receptors. In the *C. albicans* model, EV decoration with O- and N-linked mannans, along with the presence of β-1,3-glucans and chitin oligomers, modulates the activation of Toll-like receptor 4 (TLR4) and dectin-1 [42]. Notably, *C. albicans* EVs—previously identified as vaccine candidates [43,44]—have been shown to induce a protective immune response that depends on TLR4 [42]. Pre-exposure of mice to *C. albicans* EVs significantly reduced the severity of keratitis, lowered fungal burden, and improved disease prognosis [45]. These findings align with earlier reports demonstrating that fungal EVs can stimulate macrophage antifungal responses [46], induce antibodies [35,37,43], activate DC-SIGN receptors [47], enhance Th1/Th17 responses [48], modulate macrophage oxidative burst [49], promote macrophage’s M1 polarization [23], and activate galectin-3-mediated antifungal responses [50,51], among other immunological effects. Taken together, this growing body of evidence strongly supports the idea that fungal EVs can benefit the host, further highlighting their potential as promising vaccine candidates.

## Do fungal EVs harm the host?

Once again yes, under certain conditions. EVs released by *C. albicans*, *Saccharomyces cerevisiae*, *C. neoformans*, and *A. fumigatus* were taken up by murine macrophages at varying rates, leading to distinct cytokine responses, activation of innate immune signaling, and immune cell recruitment. Remarkably, cell wall components on *C. neoformans* and *A. fumigatus* EVs impaired efficient macrophage internalization and suppressed innate immune activation [52].

In the *Paracoccidioides brasiliensis* model, subcutaneous treatment of mice with EVs prior to infection with a virulent fungal isolate exacerbated disease, likely due to increased production of TNF-α, IFN-γ, IL-6, and MCP-1 in the lungs of infected animals [53]. Similarly, *Sporothrix brasiliensis* EVs were associated with increased fungal burden in experimental sporotrichosis [54]. In line with these findings, *C. neoformans* mutants with reduced EV production and attenuated virulence regained their pathogenicity when supplemented with EVs from wild-type cells [55].

This question stands in contrast to the previous discussion on the immunoprotective effects of fungal EVs, highlighting their potential to either enhance or suppress antifungal immune responses. These seemingly contradictory effects likely depend on the fungal species, EV composition, and experimental conditions used to stimulate host cells. However, one undeniable observation remains: fungal EVs can have paradoxical effects, underscoring the need for further immunological investigation.

## Perspectives

The future of fungal EV research has been extensively discussed in recent reviews, so we will not revisit those details here. However, there are key perspectives worth emphasizing. This is an expanding field, exploring cellular structures with immense biological potential, yet critical gaps remain. We still lack a comprehensive understanding of EV biogenesis, require more refined isolation protocols, and need clearer insights into their immunological functions, among other pressing questions. The recent influx of new research groups into this field has already accelerated progress, but it is not enough: there is still much to uncover. Join us in unraveling the mysteries of fungal EVs and help push this field forward, because neglected diseases deserve better, and science can do more.
